# The ion channel, TRPM2, contributes to the pathogenesis of radiodermatitis

**DOI:** 10.1007/s00411-018-0769-y

**Published:** 2018-11-27

**Authors:** Anne-Laure Perraud, Deviyani M. Rao, Elizabeth A. Kosmacek, Aleksandra Dagunts, Rebecca E. Oberley-Deegan, Fabienne Gally

**Affiliations:** 1 Department of Biomedical Research, National Jewish Health, 1400 Jackson St., Room K827, Denver, CO 80206 USA; 2 Department of Immunology and Microbiology, University of Colorado Denver, Denver, USA; 3 Department of Biochemistry and Molecular Biology, University of Nebraska Medical Center, Omaha, USA

**Keywords:** Radiodermatitis, Inflammation, Mouse, Transient receptor potential channels (TRP channels), Trpm2

## Abstract

Radiodermatitis is a painful side effect for cancer patients undergoing radiotherapy. Irradiation of the skin causes inflammation and breakdown of the epidermis and can lead to significant morbidity and mortality in severe cases, as seen in exposure from accidents or weapons such as “dirty bombs” and ultimately leads to tissue fibrosis. However, the pathogenesis of radiodermatitis is not fully understood. Using a mouse model of radiodermatitis, we showed that the Transient Receptor Potential Melastatin 2 (TRPM2) ion channel plays a significant role in the development of dermatitis following exposure to ionizing radiation. Irradiated TRPM2-deficient mice developed less inflammation, fewer severe skin lesions and decreased fibrosis when compared to wild type mice. The TRPM2-deficient mice also showed a faster recovery period as seen by their increased weight gain post irradiation. Finally, TRPM2-deficient mice exhibited lower systemic inflammation with a reduction in inflammatory cytokines present in the serum. These findings suggest that TRPM2 may be a potential therapeutic target for reducing the severity of radiodermatitis.

## Introduction

Radiation therapy is commonly used to treat several types of cancer (Cooperberg et al. 2010; Heminger et al. 2006; Monyak and Levitt 1989; Thomas 1993). However, the major side effect of radiation therapy is skin tissue damage, also known as radiodermatitis, which occurs in 95% of cancer patients who receive radiation therapy (Salvo et al. 2010). Radiodermatitis can become so severe that cancer treatment is halted until the skin heals which can compromise the effectiveness of treatment. While acute inflammation can be seen within hours of radiation treatment, radiodermatitis takes multiple weeks to develop and its severity progresses over time to erythema, dry or wet desquamation or ulceration. The appearance of these lesions depends on the radiation dose used for treatment as well as biological factors pertaining to the patient, including leukocyte recruitment, release of reactive oxygen species, proteases and other toxic molecules that damage the surrounding tissues. Inflammation is a complex process and contribution to tissue damage and radiodermatitis needs to be better understood.

TRPM2, a regulator of innate immunity and inflammation, is a cationic channel that is activated under conditions of oxidative stress (Knowles et al. 2013; Takahashi et al. 2011). TRPM2 belongs to the family of transient receptor potential (TRP) ion channels. It is referred to as a “chanzyme” because it represents the unique fusion of a Ca^2+^-permeable pore with an enzymatic region that exhibits residual hydrolase activity toward ADP-ribose (ADPR) (Perraud et al. 2001; Sano et al. 2001). The channel is gated by ADPR (Perraud et al. 2001), which can be produced following NAD depletion in response to radiation-induced oxidative stress. Cells expressing TRPM2 have been found to exhibit an H_2_O_2_-induced Ca^2+^-influx that was absent in cells lacking the channel (Hara et al. 2002; Perraud et al. 2005). Because TRPM2 is permeable to the universal second messenger Ca^2+^, its expression could result in altered signaling events and inflammatory responses as a result of radiation. Multiple studies have documented the role of TRPM2 in exacerbating cytokine production (Chung et al. 2015; Gally et al. 2018; Ham et al. 2012).

Although radiation-induced skin damage is well known, the mechanisms that cause this reaction are poorly understood. In the present study, we have evaluated the contribution of TRPM2 to radiodermatitis, including irradiated skin damage, lesions and weight loss, and have attributed these responses to increased production of inflammatory mediators.

## Materials and methods

### Mice

Wild-type C57BL/6 and TRPM2^−/−^ (previously described in Knowles et al. 2011) male mice were maintained in our colony and housed under pathogen-free conditions at the National Jewish Health Biological Resource Center. All mice were fed a standard rodent diet and used at 8–12 weeks of age. Experiments were carried out with National Jewish Health Institutional Animal Care and Use Committee (IACUC) approval.

### Radiation

Mice were anesthetized by i.p. injection of Ketamine/Xylazine (80–10 mg/kg), placed in an X-ray irradiator box and shielded with lead so that only the lower pelvic region was exposed to radiation for five consecutive days at a dose of 8 Gy/day. This dose regimen was chosen because it mimics the radiation therapy regimen of a patient being treated for pelvic cancers (van der Wielen et al. 2009). The mouse weight and skin lesions were documented once a week for 12 weeks.

### Clotrimazole and vehicle treatments

Clotrimazole (CTZ) was purchased from Sigma-Aldrich (cat # C6019). CTZ was first solubilized in ethanol (50 mg/ml), further diluted in corn oil to reach a 1% CTZ containing solution, and filter sterilized using membranes of 0.22 µm nominal pore size. Wild-type C57BL/6 male mice were treated topically with this solution. CTZ is clinically very well characterized as it is used as an anti-fungal agent, and its topical application is not toxic. The solution (or ethanol/corn oil as a vehicle control) was applied twice a week on affected skin areas starting as soon as lesions would appear (4 weeks after irradiation).

### Skin evaluation

**Fig. 1:**
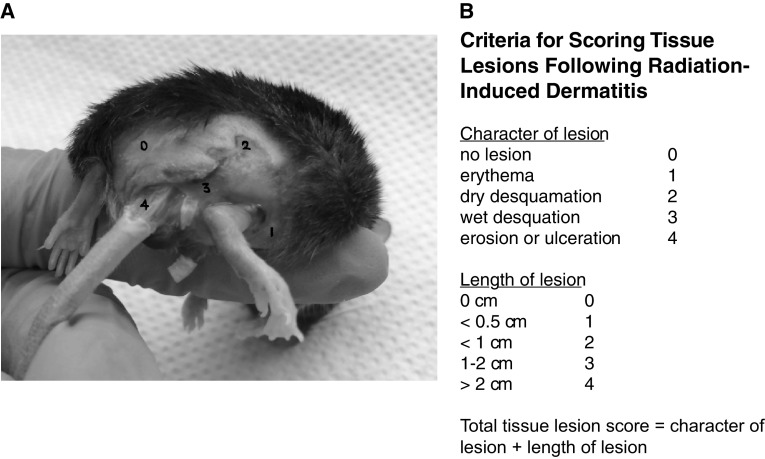
Criteria for scoring skin tissue lesions following radiation-induced dermatitis. **a** Representative picture showing the four different effects of radiation on mouse skin (adapted from (Cox et al. 1995)). **b** The scoring of the tissue lesions was obtained by adding the character of the lesion to the length of the lesion

The skin lesions were recorded and scored 12 weeks after irradiation for quality as described in (E.J. 2012) (0) normal, (1) erythema, (2) dry desquamation, (3) wet desquamation, and (4) ulceration (image shown in Fig. 1) and length of lesion: (0) 0 cm (1) < 0.5 cm, (2) < 1 cm, (3) 1–2 cm, and (4) > 2 cm.

### Trichrome staining

Mice were euthanized 12 weeks after irradiation and skin samples were obtained. Tissue specimens were fixed in 10% formalin and embedded in paraffin. Tissue sections were deparaffinized in xylenes and rehydrated in graded alcohols, then rinsed in running deionized water. Tissues were re-fixed in Bouin’s solution for 16 h at room temperature, and then rinsed for 10 min in running tap water. Nuclei were stained for 10 min in Weigert’s iron hematoxylin, and thoroughly rinsed in running warm tap water for 10 min. Muscle, cytoplasm and keratin were stained in Biebrich Scarlet-Acid Fuchsin (0.9–0.1%) for 5 min and rinsed before differentiating 15 min in 2.5% phosphomolybdic-2.5% phosphotungstic acid. Finally, collagen was stained for 8 min in 2.5% aniline blue, rinsed, and differentiated for 1 min in 1% acetic acid. Sections were dehydrated through graded alcohols, cleared in xylenes, and mounted with Permount (Fisher Scientific, cat. SP15).

### Quantification for trichrome staining

Trichrome stained sections were imaged in brightfield mode, with a 20× objective, on a Leica DM4000 B LED microscope (Leica Microsystems, Wetzlar, Germany). To measure the collagen density in the skin, each section was imaged over the length of the section requiring ten evenly spaced fields of view. Using ImageJ software, the region of interest (the dermis, excluding hair follicles, sweat glands, blood vessels, and pockets of red blood cells) was selected so that only the area containing collagen was included in the analysis. Next, thresholding was used to select only blue pixels (collagen) and excluded purple/red pixels (immune cells and keratin); white hues were excluded to eliminate holes in the tissue. The collagen density was calculated as the number of pixels representing collagen divided by the total number of pixels in the region of interest (ROI). The percent area of tissue comprised of collagen was averaged for each animal and the mean per group reported.

### Quantification for epidermal thickness

The epidermal layer thickness was quantified using the trichrome staining images. For each image, approximately 20 equally spaced measurements were made along the length of the tissue by drawing a line from the junction of the dermis and epidermis to the edge of the epithelial layer. The pixel value was converted to microns using a factor of 3.84 pixels/micron. A mean epidermal thickness was calculated for each animal using all images containing epithelium.

### Cytokine measurement

Serum was separated using Z-Gel microtubes (Sarstedt), clotting blood for 30 min at room temperature, and serum removed by centrifugation (8000 rpm for 5 min). Meso Scale Discovery V-Plex Proinflammatory Panel 1 (mouse) plates were used according to the manufacturer’s instructions.

### Immunostaining

Tissue sections were deparaffinized in xylenes and rehydrated in graded alcohols, then rinsed in running deionized water. Antigen retrieval was performed by boiling slides in 10 mM sodium citrate buffer, pH 6.0 for 20 min, followed by a 20-min cool down, and a 10-min PBS wash. Endogenous peroxidases were quenched for 5 min in 3% H_2_O_2_ in PBS, followed by a 5-min wash. Next, slides were blocked in 10% goat serum for 30 min followed immediately by 1 h incubation in primary antibody. Primary antibodies included CD68 (1:100, Abcam, cat. ab125212), CD3 (1:100, Abcam, cat. ab5690), and TRPM2 (1:1000, Abcam, cat. ab11168). Negative stain controls were incubated in blocking buffer without primary antibody for 1 h. Following several washes in PBS, the sections were next incubated in biotinylated goat anti-rabbit secondary antibody (1:200, Vector Labs, cat. BA-1000) then washed in PBS again. Peroxidase activity was associated to the biotinylated secondary antibody using the Vector Labs ABC Kit (cat. PK-4000) by incubation for 30 min in ABC buffer. Finally, DAB substrate was applied to detect the proteins of interest (Vector Labs, cat. SK-4100) for 5–7 min until the brown color was visible under a microscope. Slides were counterstained by briefly dipping in Harris hematoxylin (Sigma-Aldrich, St. Louis, MO, USA, cat. HHS16), then dehydrated through graded alcohols, cleared in xylenes, and mounted with Permount solution.

For both CD3 and CD68 in skin, six random fields of view were captured with a 20× objective, on a Leica DM4000 B LED microscope. Cells staining a deep brown color were manually counted using the “multi-point” function in ImageJ software. The average cells per field were reported and used for statistical analysis. For TRPM2, serial sections were stained for TRPM2, CD68 and CD3 to determine if TRPM2 expression co-localized in lymphocytes and macrophages.

### Statistical analysis

Data are expressed as mean ± SEM. One-way analysis of variance was used for multiple comparisons, and Tukey’s post hoc test was applied where appropriate. Student’s *t* test was used when only two groups were compared. Differences were considered statistically significant when *p* < 0.05.

## Results

**Fig. 2:**
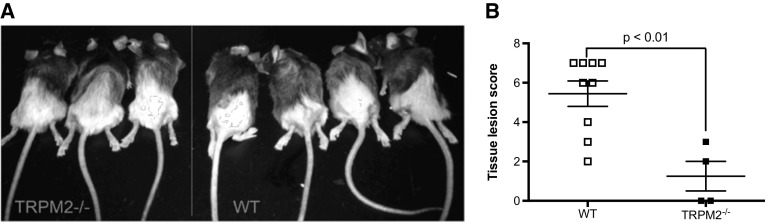
Radiation-induced dermatitis is reduced in TRPM2^−/−^ mice. **a** Representative photo images of irradiated WT and TRPM2^−/−^ mice 12 weeks post irradiation. **b** Severity of the lesions was quantified using the scoring system described in Fig. 1 on a scale from 0 to 8. *N* = 5–9 mice per group

Wild-type (WT) and TRPM2^−/−^ male mice were irradiated for five sequential days with 8 Gy/day in the pelvic region. This radiation scheme was used previously in rats (Oberley-Deegan et al. 2012) to mimic what a patient with a cancer in the pelvic region would undertake with radiation therapy (van der Wielen et al. 2009). At 2 weeks post irradiation, WT mice started to show signs of hair loss and skin erythema in the exposed pelvic region. At 4 weeks, WT mice developed a pronounced loss of hair along with erythema and desquamation of the epidermis while TRPM2^−/−^ mice had just begun to show signs of hair loss. At 12 weeks, while TRPM2^−/−^ mice had fully recovered from hair loss, WT mice still exhibited erythema and desquamation (Fig. 2A). Using the scoring system as described in Fig. 1, we determined that TRPM2^−/−^ had significantly less skin lesions than WT mice 12 weeks post irradiation (Fig. 2B).

**Fig. 3:**
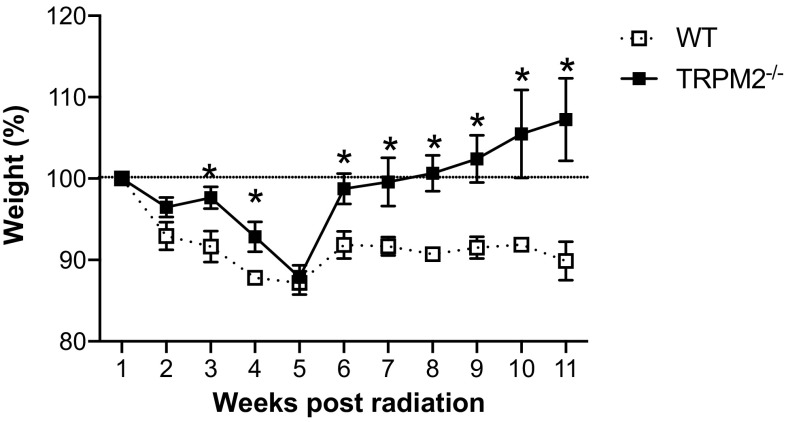
Weight loss is reduced in TRPM2^−/−^ mice following irradiation. Weights of irradiated animals throughout the course of the experiment. *N* = 5–9 mice per group, *p < 0.05

Weight loss is a well-documented and common side effect from irradiation that can indicate the severity of patient’s reaction to radiation therapy which leads us to weigh the mice weekly throughout the 12-week experiment (Fig. 3). While there were no differences in weight loss between WT and TRPM2^−/−^ mice up to 5 weeks post-irradiation, TRPM2^−/−^ mice recovered and gained weight steadily from 6 to 12 weeks post radiation whereas the WT mice never recovered from their initial weight loss (Fig. 3).

**Fig. 4:**
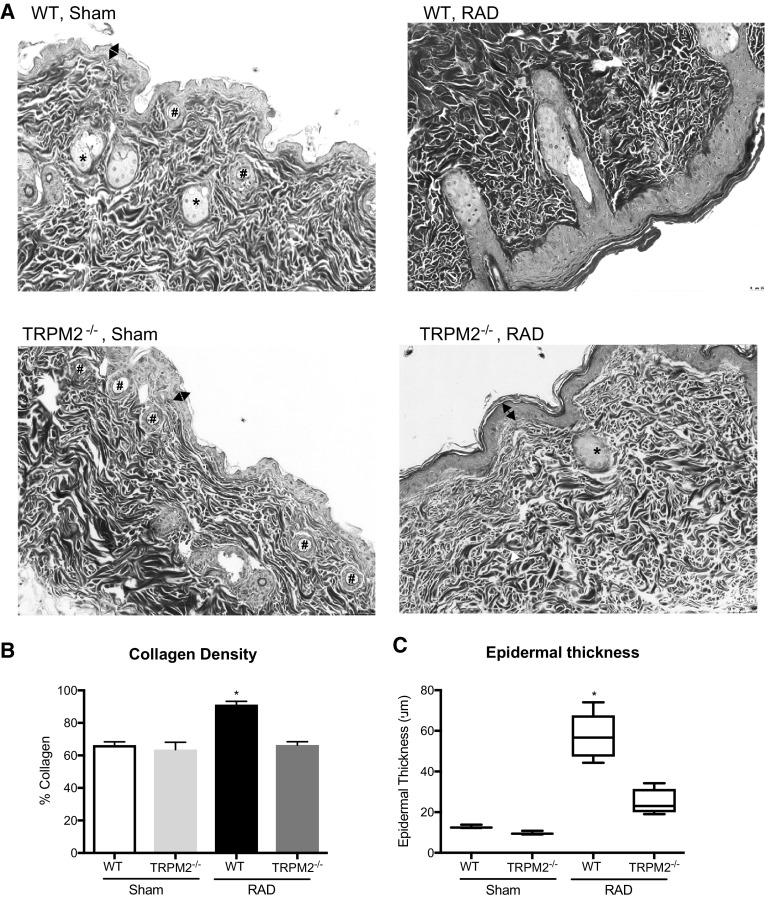
Radiation-induced skin fibrosis and epidermal thickening is reduced in TRPM2^−/−^ mice. **a** Representative images of trichrome stained WT and TRPM2^−/−^ sham and lesional skin 12 weeks post irradiation. Stars indicate sebaceous glands, pounds indicate hair follicles, white arrows indicate inflammatory cells, double arrows indicate the epidermis. Collagen density is proportionate to the intensity of the blue stain. **b** Collagen quantification using trichrome staining. **c** Average epidermal thickness

At 12 weeks post irradiation, we performed histological analysis of the skin tissue. Un-irradiated (sham) WT or TRPM2^−/−^ skin did not show any obvious differences (Fig. 4a, left panels). Representative images of lesional irradiated skin regions show that the WT mouse skin appeared more fibrotic and infiltrated with inflammatory cells as compared to TRPM2^−/−^ mice (Fig. 4a, right panels). Next, we quantified the fibrosis of the skin by measuring the amount of collagen present using trichrome staining and observed significantly higher levels of collagen in irradiated WT mice as compared to irradiated TRPM2^−/−^ mice, while un-irradiated WT or TRPM2^−/−^ skin showed no difference (Fig. 4b). We also analyzed alterations in epidermal thickness and measured an increase in irradiated WT mice as compared to irradiated TRPM2^−/−^ mice, while un-irradiated WT or TRPM2^−/−^ skin showed no difference (Fig. 4c).

**Fig. 5:**
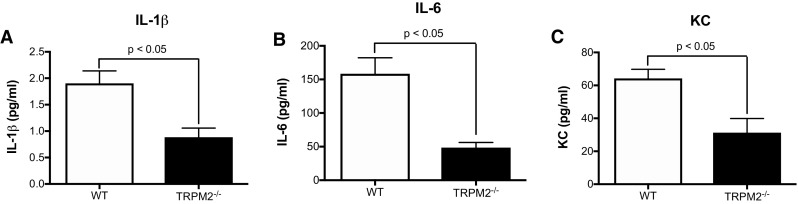
Inflammatory cytokines are reduced in TRPM2^−/−^ serum. **a** IL-1β, **b** IL-6, **c** KC. *N* = 5–9 mice per group

**Fig. 6:**
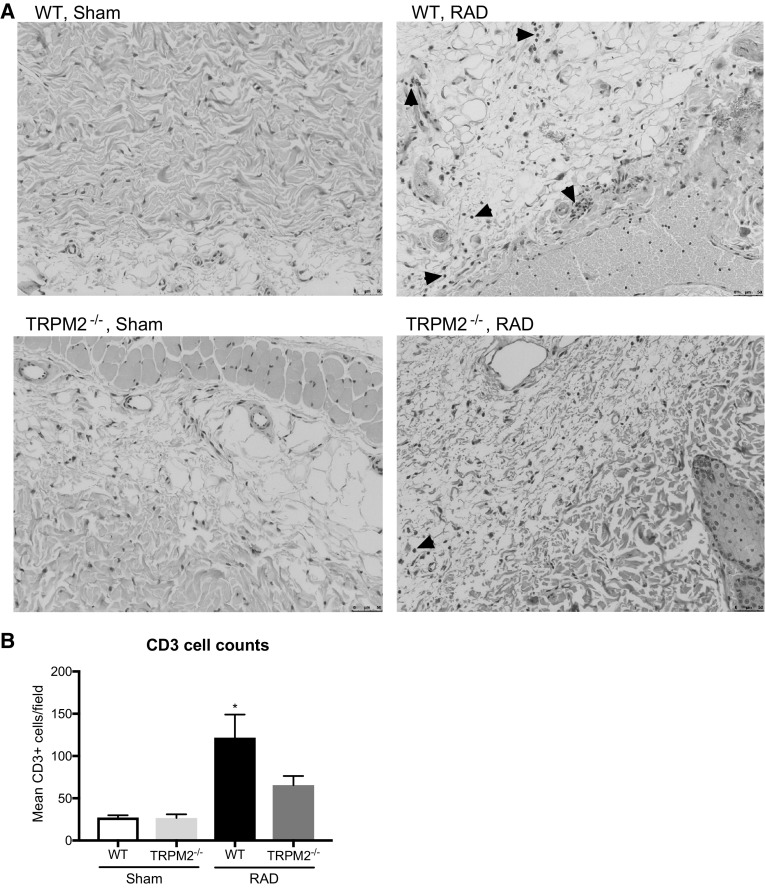
Radiation-induced T cell infiltration is reduced in TRPM2^−/−^ mice. **a** Representative images of CD3 stained WT and TRPM2^−/−^ sham and lesional skin 12 weeks post irradiation. Arrowheads indicate CD3+ cells. **b** Quantification of CD3 cell numbers per field

**Fig. 7:**
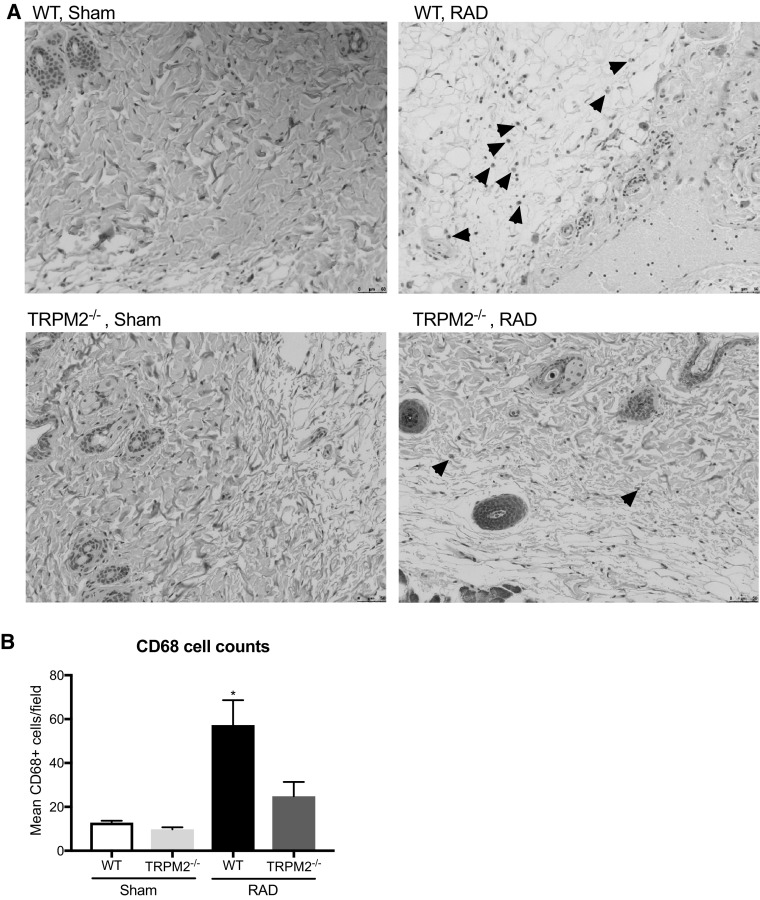
Radiation-induced macrophage infiltration is reduced in TRPM2^−/−^ mice. **a** Representative images of CD68 stained WT and TRPM2^−/−^ sham and lesional skin 12 weeks post irradiation. Arrowheads indicate CD68+ cells. **b** Quantification of CD68 cell numbers per field

As radiation is known to elicit an inflammatory response, we measured the systemic inflammation in the serum of both WT and TRPM2^−/−^ mice 4 weeks post irradiation. In accordance with our previous observations, TRPM2^−/−^ mice had reduced inflammatory cytokines, including IL-1β, IL-6 and KC as compared to WT mice (Fig. 5). Additionally, we quantified the amount of lymphocytes (CD3+) and macrophages (CD68+) infiltrating cells in the skin tissue (Figs. 6, 7) 12 weeks post irradiation. While un-irradiated WT or TRPM2^−/−^ skin showed no difference in the amount of lymphocytes or macrophages at baseline, irradiated WT skin showed a significant increase in both, lymphocytes and macrophages, as compared to irradiated TRPM2^−/−^ skin. Taken together, these results suggest that TRPM2-deficiency may play a protective role in radiation-induced damage in part by inhibiting systemic inflammation and leukocyte recruitment.

**Fig. 8:**
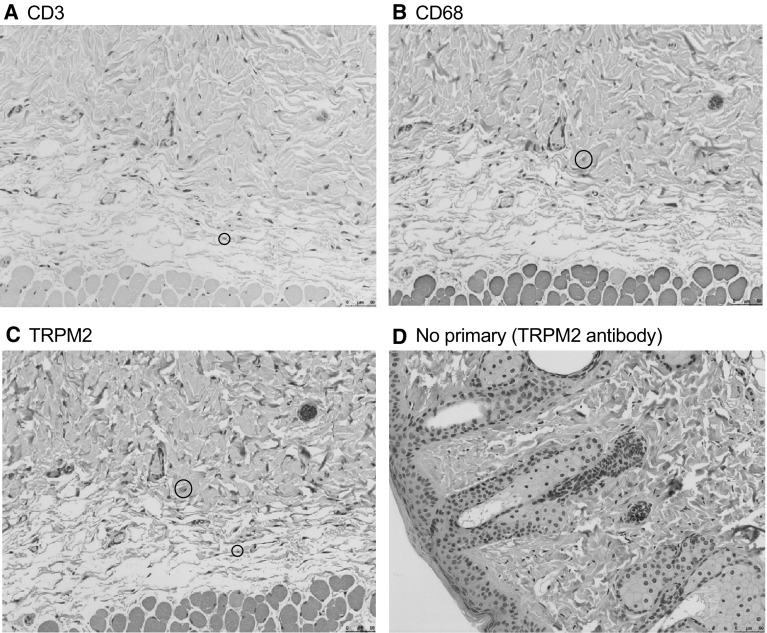
Infiltrating immune cells express TRPM2. Representative images of irradiated WT skin stained with **a** CD3, **b** CD68, **c** TRPM2, **d** no primary TRPM2 antibody (negative control). Circles indicate double positive cells for either CD3 or CD68 and TRPM2 staining

To further demonstrate that TRPM2 is implicated in radiation-induced inflammation, we stained serial sections of irradiated WT skin tissue for CD3, CD68 and TRPM2 (Fig. 8). Both CD3 positive cells and CD68 positive cells are also positive for TRPM2. These data demonstrate that recruited T lymphocytes and macrophages following radiation express TRPM2.

**Fig. 9:**
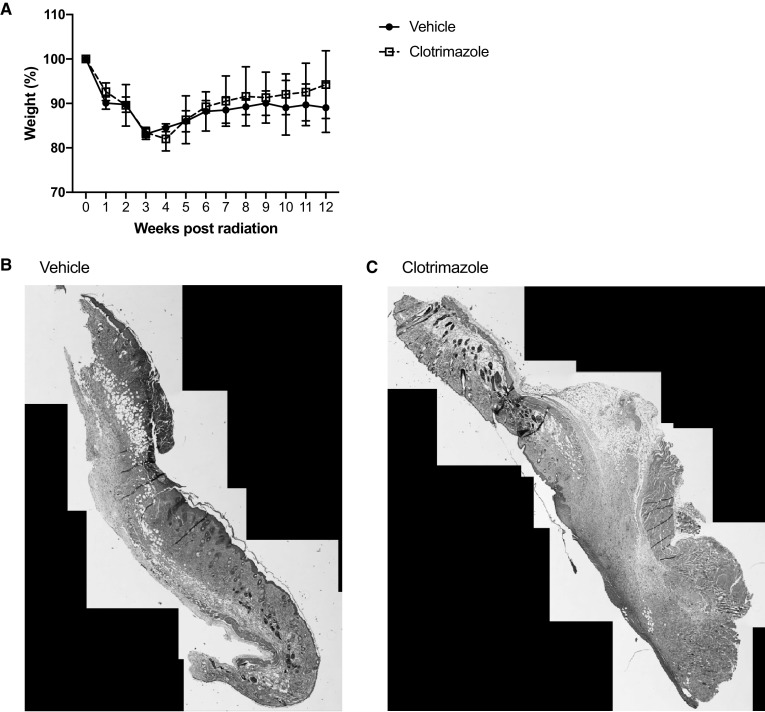
Apical TRPM2 inhibition did not protect against radiation-induced weight loss and dermatitis. **a** Weights of WT irradiated animals treated with vehicle or clotrimazole throughout the course of the experiment. *N* = 5 mice per group. Representative stitched images of irradiated WT skin treated with **b** vehicle or **c** clotrimazole. Data are representative of two independent experiments

To test whether local administration of TRPM2 inhibitors is sufficient to protect against radiodermatitis, we administered a topical TRPM2 inhibitor (clotrimazole) following irradiation of WT mice. As illustrated in Fig. 9a, mice that received clotrimazole lost as much weight as mice that received vehicle treatment. Furthermore, stitched images, using FIJI, of lesional skin showed no difference between vehicle or clotrimazole treatment (Fig. 9b). Since immune cells require systemic blockade that is not provided by the apical treatment, these data further confirm the implication of TRPM2-induced immune cell recruitment and inflammation.

## Discussion

In this study, we have demonstrated that TRPM2-deficiency decreases the severity of various side effects associated with radiation exposure. Specifically, we have shown that TRPM2^−/−^ mice are protected from skin damage and overall weight loss associated with lower abdominal radiation exposure. Furthermore, histological analysis of skin lesions showed that TRPM2-deficiency protected the tissue from irradiation-induced damage by limiting the inflammation and the development of fibrosis in irradiated skin. Finally, we showed that TRPM2^−/−^ mice had significantly lower circulating inflammatory cytokines and lower leukocyte recruitment, but apical inhibition of TRPM2 had no effect on radiation-induced dermatitis. Taken together, these data suggest that TRPM2 deficiency is protective against radiation-induced skin damage and helps preserve the function of this organ.

The mechanism by which TRPM2-deficiency is most likely protecting the irradiated skin from damage is by decreasing inflammation at the site of exposure. In our studies, radiation-induced TRPM2^−/−^ skin lesions showed less infiltration of inflammatory cells as well as decreased levels of systemic inflammatory cytokines, specifically IL-1β, IL-6 and KC. TRPM2 is known to promote inflammation and cytokine production in various situations (Gally et al. 2018; Perraud et al. 2004; Syed Mortadza et al. 2015). Thus, inhibiting TRPM2 may reduce the severity of radiodermatitis by dampening inflammation systematically and thus halting the vicious cycle of chronic immune activation and tissue injury.

Alternatively, since radiogenic TRPM2 activation and involvement of TRPM2 in DNA damage response has previously been reported (Klumpp et al. 2016; Masumoto et al. 2013), TRPM2 in the skin might boost immunogenic cell death. While TRPM2 in immune cells would require systemic blockage, local administration of TRPM2 inhibitors would be sufficient to protect against radiation-induced TRPM2 activation and DNA damage. We, thus, administered clotrimazole, a known TRPM2 inhibitor (Hill et al. 2004b), locally to the skin lesions. Clotrimazole did not improve the outcome of radiation-induced dermatitis, thus confirming the importance of TRPM2-induced immune activation.

Ionizing radiation triggers the activation of keratinocytes, fibroblasts and endothelial cells to secrete pro-inflammatory cytokines such as IL-1β, IL-6 and KC (Ryan 2012). In turn, IL-1β could activate γδ T cells and induce IL-17 expression leading to a pathogenic inflammatory response (Liao et al. 2017). Interestingly, the IL-1 pathway has been shown to play a significant role in the development of radiodermatitis (Janko et al. 2012). Mice lacking IL-1 or IL-1 receptor have a decrease in inflammation and pathological changes to their skin, similar to what we observed for the TRPM2^−/−^ mice (Janko et al. 2012). IL-1 is one of only few cytokines that is induced after skin irradiation and has been implicated in chronic radiodermatitis-induced fibrosis (Liu et al. 2006). The reduced IL-1β production that we observed in TRPM2^−/−^ mice may therefore be sufficient to protect them from radiodermatitis.

Our findings may have relevance for radiation injury in other tissues since we measured increased levels of inflammatory cytokines in the periphery. TRPM2 was previously found to contribute to irreversible loss of salivary gland function following irradiation, which is a severe side effect of radiotherapy for head and neck cancers (Liu et al. 2013). In a follow-up study, it was shown that TRPM2 functions as an important regulator of salivary glands, further supporting the utility of targeting TRPM2 to protect a wide range of tissues against radiation-mediated injury (Liu et al. 2017).

Several compounds have been shown to inhibit TRPM2 currents. For instance, as stated previously, we used clotrimazole to see if we could prevent radiation-induced skin injury by apically blocking TRPM2. Other compounds such as 2-aminoethoxydiphenyl borate (Togashi et al. 2008) and the anti-fungal econazole (Hill et al. 2004b) have been shown to inhibit ADP-ribose activated TRPM2 currents. Flufenamic acid, a nonsteroidal anti-inflammatory drug, is another TRPM2 inhibitor (Hill et al. 2004a) but it is difficult to dissolve which might be problematic for use at high concentrations. *N*-(*p*-amylcinnamoyl)anthranilic acid inhibits TRPM2 (Kraft et al. 2006), but it also functions as a phospholipase A_2_ inhibitor (Chen et al. 1994). Our studies suggest that a systemic inhibition of TRPM2 would be required to alleviate the effects of radiation on skin damage.

Radiodermatitis is a serious side effect due to radiotherapy to treat many types of tumors found throughout the body, which can lead to the delay of therapeutic treatments. Furthermore, the skin is the first organ that would be affected in a nuclear accident or “dirty bomb” detonation and as such exposed to whole body irradiation. However, given that our understanding of the inflammatory pathways involved in radiodermatitis is still limited, we currently do not have an effective treatment for controlling damage to the skin. Our results emphasize the importance of TRPM2 in mediating radiation-induced inflammatory responses and suggest TRPM2 as a potential target when considering therapeutic interventions for radiodermatitis.
